# Mapping language function with task-based vs. resting-state functional MRI

**DOI:** 10.1371/journal.pone.0236423

**Published:** 2020-07-31

**Authors:** Ki Yun Park, John J. Lee, Donna Dierker, Laura M. Marple, Carl D. Hacker, Jarod L. Roland, Daniel S. Marcus, Mikhail Milchenko, Michelle M. Miller-Thomas, Tammie L. Benzinger, Joshua S. Shimony, Abraham Z. Snyder, Eric C. Leuthardt

**Affiliations:** 1 Mallinckrodt Institute of Radiology, Washington University School of Medicine, St. Louis, Missouri, United States of America; 2 Department of Neurosurgery, Washington University School of Medicine, St. Louis, Missouri, United States of America; 3 Department of Neurosurgery, University of California San Francisco, San Francisco, California, United States of America; University of North Carolina at Chapel Hill, UNITED STATES

## Abstract

**Background:**

Use of functional MRI (fMRI) in pre-surgical planning is a non-invasive method for pre-operative functional mapping for patients with brain tumors, especially tumors located near eloquent cortex. Currently, this practice predominantly involves task-based fMRI (T-fMRI). Resting state fMRI (RS-fMRI) offers an alternative with several methodological advantages. Here, we compare group-level analyses of RS-fMRI vs. T-fMRI as methods for language localization.

**Purpose:**

To contrast RS-fMRI vs. T-fMRI as techniques for localization of language function.

**Methods:**

We analyzed data obtained in 35 patients who had both T-fMRI and RS-fMRI scans during the course of pre-surgical evaluation. The RS-fMRI data were analyzed using a previously trained resting-state network classifier. The T-fMRI data were analyzed using conventional techniques. Group-level results obtained by both methods were evaluated in terms of two outcome measures: (1) inter-subject variability of response magnitude and (2) sensitivity/specificity analysis of response topography, taking as ground truth previously reported maps of the language system based on intraoperative cortical mapping as well as meta-analytic maps of language task fMRI responses.

**Results:**

Both fMRI methods localized major components of the language system (areas of Broca and Wernicke) although not with equal inter-subject consistency. Word-stem completion T-fMRI strongly activated Broca's area but also several task-general areas not specific to language. RS-fMRI provided a more specific representation of the language system.

**Conclusion:**

We demonstrate several advantages of classifier-based mapping of language representation in the brain. Language T-fMRI activated task-general (i.e., not language-specific) functional systems in addition to areas of Broca and Wernicke. In contrast, classifier-based analysis of RS-fMRI data generated maps confined to language-specific regions of the brain.

## Introduction

Localizing the representation of language in the brain has significant clinical utility in the identification of eloquent cortex prior to neurosurgical procedures [1–3]. Before the advent of non-invasive functional neuroimaging, direct stimulation of the cortical surface was the only means of accomplishing this objective [4–7]. More recently, non-invasive pre-operative localization has been achieved using functional magnetic resonance imaging (fMRI), most commonly using task-based protocols [8, 9]. Pre-surgical task-based fMRI (T-fMRI) mapping of language function is commonly used in appropriate cases [10].

Resting state fMRI (RS-fMRI) [11] is an alternative method for localizing the representation of function in the brain. RS-fMRI delineates topographies associated with specific functions, e.g., somatomotor, executive control, language, etc. These topographies are widely known as resting state networks (RSNs) [12, 13]. RS-fMRI offers potential advantages in comparison to T-fMRI: Patients are not required to perform any task other than limiting head motion during scanning. Thus, the behavioral protocol is maximally simple ("hold still and try to stay awake") and no ancillary task-related apparatus, e.g., MR-compatible display systems, is required. Moreover, RS-fMRI is compatible with light sedation or sleep and is feasible even in young children [14, 15]. Notwithstanding these potential advantages, it remains uncertain whether the functional maps obtained by T-fMRI and RS-fMRI are comparable. This is the question addressed here.

It is widely recognized that RSNs derived by analysis of RS-fMRI data in healthy subjects exhibit topographic similarities with task-evoked responses [16]. This similarity is especially clear in primary sensory and motor systems [17–19]. Therefore, it is not surprising that T-fMRI and RS-fMRI are comparably effective in localizing somato-motor cortex in the context of presurgical functional mapping [20–22]. However, somato-motor function is simply (somatotopically) organized within one contiguous area of the cerebral cortex [23]. In contrast, higher order cognitive operations do not simply map onto RSNs [24–26]. Language, in particular, encompasses multiple separable faculties, e.g., semantic decoding/encoding, motor speech, etc., and is represented in several topologically distinct areas of the brain [27–29].

In view of these complexities, we pursued a data-driven comparison of T-fMRI vs. RS-fMRI localization of language. We depart from prior work on this topic in two important respects. First, prior work is largely formulated in terms of within-patient comparisons; this is appropriate when the objective is to determine which method best localizes function in an individual prior to neurosurgery. However, in current clinical practice, the total duration of fMRI scanning typically is on the order of 6 minutes per condition (for reviews see [10, 30]) whereas considerably longer acquisitions are required to obtain reliable RS-fMRI results in individuals [31, 32]. Thus, conventionally acquired individual fMRI results, RS-fMRI as well as T-fMRI, necessarily are signal-to-noise ratio (SNR) limited [33]. Averaging over subjects enhances the SNR. Accordingly, we conducted group-level analyses with the objective of revealing features at the population level not evident in conventional single subject results. Second, the extant literature is dominated by spatial independent component analysis (sICA) of RS-fMRI data [34]. We analyzed the RS-fMRI data using a previously trained multi-layer perceptron (MLP) [28]. MLP-based pre-surgical functional localization has performed well in several case series [14, 15, 20, 35, 36]. MLP analysis eliminates operator-dependent steps, e.g., selection of ICs. Additionally, the MLP is a supervised (as opposed to unsupervised) classifier, hence offers high sensitivity and specificity in comparison to alternative methods [28]. Direct comparison of MLP vs. sICA of RS-fMRI data is of interest but will be reported separately.

Our analyses address the following questions: (1) Are the whole-brain maps obtained by T-fMRI and RS-fMRI topographically similar on average? (2) Are the responses generated by T-fMRI and RS-fMRI comparably consistent over individuals? (3) Do the maps obtained by T-fMRI and RS-fMRI similarly overlap language regions of interest (ROIs) determined by *a priori* methods? We address these questions using group-level analyses of data acquired in 35 patients with brain tumors.

## Methods

### Patient identification

Patients were retrospectively identified through the Neurosurgery brain tumor service, initially as part of an NIH-funded tumor data base grant (CONDR NIH 5R01NS066905). Patients were drawn from the same data base reported in a prior study targeting non-invasive localization of sensorimotor cortex [20]. All data was anonymized and was accessed from the medical records of Barnes-Jewish Hospital in St. Louis, Missouri during August 2015 from scans done during 2014. All MRIs were the first done after diagnosis in preparation for surgery. All patients who had both language task (word-stem completion) fMRI and RS-fMRI prior to neurosurgery were included in the present analysis. We identified N = 35 (23 male and 12 female) patients (age range 23–71 years; mean, 44.8 years). The mean preoperative tumor volume was 43.8mL (range: 1.4–207 mL); 28 patients had a left-hemisphere tumor; pathology was most often oligoastrocytoma (11 cases) and glioblastoma (10 cases). Handedness was recorded in 26; 23/26 patients were right-handed. Patient demographics are summarized in Table 1. All aspects of the study were approved by the Institutional Review Board at Washington University School of Medicine in St Louis. Clinical data were acquired during preoperative evaluation and reviewed retrospectively.

**Table 1:** Patient clinical and demographic data.

Patient ID (N = 35)	Age (yrs)	Sex	Handedness	Tumor Location	Tumor Size (mL)	Tumor Pathology
RS_003	44	M	R	Left basal ganglia	8.7	Glioblastoma
				Left temporal lobe	4.8	
RS_004	24	M	R	Left frontal lobe	56.2	Anaplastic glioma
RS_005	36	M	NA	Left frontal lobe	1.2	Anaplastic mixed oligoastrocytoma
				Left frontal lobe	0.2	
RS_006	36	M	NA	Left inferior frontal lobe	81.1	Anaplastic mixed oligoastrocytoma
RS_007	64	M	R	Left parieto-occipital	85.1	Glioblastoma,
RS_009	65	F	R	Left peri-trigonal area	147	Glioblastoma
RS_011	24	M	R	Left frontotemporal	56.4	Mixed oligoastrocytoma
RS_012	42	M	R	Left frontal lobe	7.8	Anaplastic oligodendroglioma
RS_014	44	M	R	Left frontal/insular lobe	69.2	Oligodendroglioma
RS_015	62	F	NA	Left frontal lobe	34.7	Mixed oligoastrocytoma
RS_016	57	F	NA	Left insula	15.2	Glioblastoma
RS_017	54	M	R	Left frontal lobe	64.3	Mixed oligoastrocytoma
RS_018	39	F	R	Left frontal lobe	13.5	Oligodendroglioma
RS_019	33	F	R	Right frontoparietal	207	Anaplastic oligodendroglioma
RS_020	53	F	R	Left temporal lobe	19.9	Glioblastoma
RS_021	25	M	R	Left frontal lobe	63.3	Mixed oligoastrocytoma
RS_022	67	M	NA	Right frontal lobe	2.2	Metastatic lung carcinoma
RS_023	50	F	R	Left parietal/splenium	28.7	Oligodendroglioma
RS_024	56	M	R	Left frontal lobe	4.7	Anaplastic oligoastrocytoma
RS_027	45	M	L	Left temporal lobe	24.8	Low-grade diffuse glioma
RS_029	52	M	R	Left frontal lobe	14.5	Oligodendroglioma
RS_030	71	M	R	Right basal ganglia/thalamus	16.6	Glioblastoma
RS_031	53	F	NA	Left thalamus	5.8	Glioblastoma
RS_032	46	M	R	Right temporal lobe	5.7	Glioblastoma
RS_033	37	M	R	Left frontal lobe	185	Mixed oligoastrocytoma
RS_034	58	F	NA	Left temporal lobe	24.9	Meningioma
RS_035	28	F	R	Left temporal lobe	10.1	Oligoastrocytoma
RS_039	25	M	L	Right parietal lobe	32.0	Mixed oligoastrocytoma
RS_040	39	F	R	Right sylvian fissure	31.5	Ependymoma
RS_041	40	M	NA	Left frontal lobe	23.3	Mixed oligoastrocytoma
RS_042	60	M	R	Left parietal lobe	0.7	Glioblastoma
RS_043	33	M	R	Right temporal lobe	4.0	Low-grade glioneuronal tumor
RS_044	23	M	R	Left frontal lobe	0.4	Ganglioglioma
RS_045	28	F	L	Bilateral frontal lobes (left>right)	118	Anaplastic astrocytoma
RS_047	55	M	NA	Left frontal lobe	66.2	Glioblastoma

Clinical data for 35 patients with brain tumors (age 44.8 ± 14.0 yrs; 12 female).

### Functional MRI acquisition

Patients were scanned with either of two 3 Tesla MR scanners (Trio or Skyra, Siemens, Erlangen, Germany) using a standard clinical pre-surgical tumor protocol. Anatomical imaging contributing to the present analyses included T1-weighted magnetization prepared rapid acquisition gradient echo (MP-RAGE) and T2-weighted fast spin echo. Both the task-based and resting-state fMRI were acquired using a T2*-weighted echo planar imaging sequence (voxel size 3×3×3mm; TE = 27ms; TR = 2.2s; field of view = 256 mm; flip angle = 90°). During T-fMRI, patients covertly generated words in response to visually presented first letter [37]. Task/rest blocks (10 frames each) were repeated over 5 off/on cycles for a total of 100 frames (3:40 minutes/T-fMRI run). RS-fMRI was acquired as two 160-frame runs (total of 320 frames = 11:44 minutes). If more than one T-fMRI run was acquired, the run with the lowest root-mean-square head motion measure was used in the present analysis. Duration of RS-fMRI acquisition was determined by time constraints of clinical MRI scanning.

### Preprocessing

We preprocessed fMRI data using previously described techniques [38] (See Supplemental Materials). Software used included the 4dfp suite (4dfp.readthedocs.io). All fMRI data acquired in each patient were pooled in the preprocessing step that compensated for head motion. Thus, the T-fMRI and RS-fMRI data were mutually co-registered. The quantity of RS-fMRI data was 3 times greater than the quantity of T-fMRI (11:44 vs. 3:40 minutes). Therefore, to equate acquisition time between RS-fMRI and T-fMRI (11:44 vs. 3:40 minutes), we divided the pre-processed RS-fMRI data into 3 equal portions. We then analyzed each portion using a previously trained multi-layer perceptron (MLP) [28].

MLPs are supervised classifiers that are trained to map input data to pre-defined output classes using hidden layers. In this work, the MLP was previously trained to associate correlation maps generated from canonical ROIs with a priori class labels corresponding to seven predefined RSNs. The MLP consisted of an input, hidden, and output node layer, fully connected in a feed-forward manner. Each training input was a correlation map generated from one of 169 canonical seed ROIs in normal control subjects. After training, the MLP was applied comprehensively to the entire brain by generating a correlation map for each voxel (treating each voxel as a seed) and then computing RSN estimates by propagating this map through the MLP. The MLP assigns to each voxel 7 values in the range [0, 1] expressing the likelihood of belonging to each of 7 RSNs.

Here, the likelihood of belonging to the LAN (language) RSN was taken as the MLP measure of language representation. T-fMRI responses were evaluated using standard general linear model methods. Activation maps were generated from the task fMRI as described in [39], smoothed with a 6mm Gaussian filter, and masked to exclude extra-cranial voxels. The MLP language RSN and task activation maps both were resampled to 1(mm)^3^ voxels prior to analysis of intersection with the *a priori* defined regions of interest (see immediately below).

### *A priori* defined language regions of interest

We defined language ROIs based on T-fMRI responses aggregated by Neurosynth [40] as well as stimulation mapping results reported in the summary paper of [5]. ROIs were confined to the left hemisphere to simplify comparison between T-fMRI-based vs. stimulation-based definitions. To define T-fMRI-based language ROIs, we queried Neurosynth using “language comprehension” as a search term (with suggested threshold Z = 3.7). Post-processing of the Neurosynth results (details in Supplemental Materials) ensured that the volumes of the Broca-like and Wernicke-like ROIs were comparable. Stimulation mapping-based ROIs were defined by a board certified neuro-radiologist (JSS) using the surface loci with positive language findings (speech-arrest, anomia, or alexia), as reported in [5] (see Supplemental Materials).

### Image computation and visualization software

fMRI preprocessing, denoising, and computation of RS-fMRI and T-fMRI responses were done with in-house software (https://readthedocs.org/projects/4dfp/). The 4dfp suite of programs is downloadable and documented on-line. AFNI 3dmean was used to create group-level mean and standard deviation volumetric images. Caret (http://brainvis.wustl.edu/wiki/index.php/Caret) used to project ROI from the PALS-B12 surface to a 3D atlas volume. Connectome Workbench (https://www.nitrc.org/projects/workbench/) was used to project the volumetric data onto the PALS-B12 mid-thickness surface, to perform algebraic operations on surface representations, and to visualize results. Statistical computations were run using MATLAB (R2017a, The MathWorks). Software versions and availability are listed in Supplemental Materials.

### Statistical analysis

Statistical analysis was performed using Matlab (v.2019b, update 5, Natick, MA) with relevant portion available in Github (github.com/jjleewustledu/mlparklang). T-fMRI and RS-fMRI yield voxelwise measures with very different statistical properties. T-fMRI responses are evaluated as percent BOLD modulation; the range of observed values is not constrained and may even include negative responses. The MLP generates volumetric maps representing the likelihood of network affiliation in the range 0 to 1 [28]. Each map generated by the current implementation of the MLP is constrained to assume a uniform distribution of values over the brain. To enable meaningful comparisons between maps obtained by T-fMRI and RS-fMRI, it was necessary to ensure that the response measures obtained by both techniques conformed to the same distribution. To this end, the measures obtained by both T-fMRI and the MLP language map were averaged over patients and the distributions of these measures, summed over the brain, were matched using the probability integral transformation. Following, response distribution matching, maps obtained by T-fMRI vs. RS-fMRI were assessed voxelwise as the signal-to-noise ratio (SNR), i.e., the group mean response divided by the standard deviation computed over patients. The divisor in the SNR includes all sources of noise in individual functional responses (electronic noise and physiologic artifact) [41]. However, the primary source of variance in the group average is individual response magnitude variability. Thus, the SNR image reflects response consistency. SNR was evaluated identically for distribution-matched T-fMRI and RS-fMRI data.

In addition to SNR maps, we assessed response topography in relation to standard language function maps, using receiver operating characteristic (ROC) curve analysis (See Supplemental Materials) Sensitivity/specificity curves parametric in response threshold were evaluated for both T-fMRI and RS-fMRI with the response distributions matched as described above. We then quantitated the match to standard topography in terms of area under the curve (AUC). Statistical analysis of the AUC results was run using the fast implementation of DeLong’s algorithm [42, 43].

## Results

### Mean RS-fMRI and T-fMRI language maps

**Fig 1:**
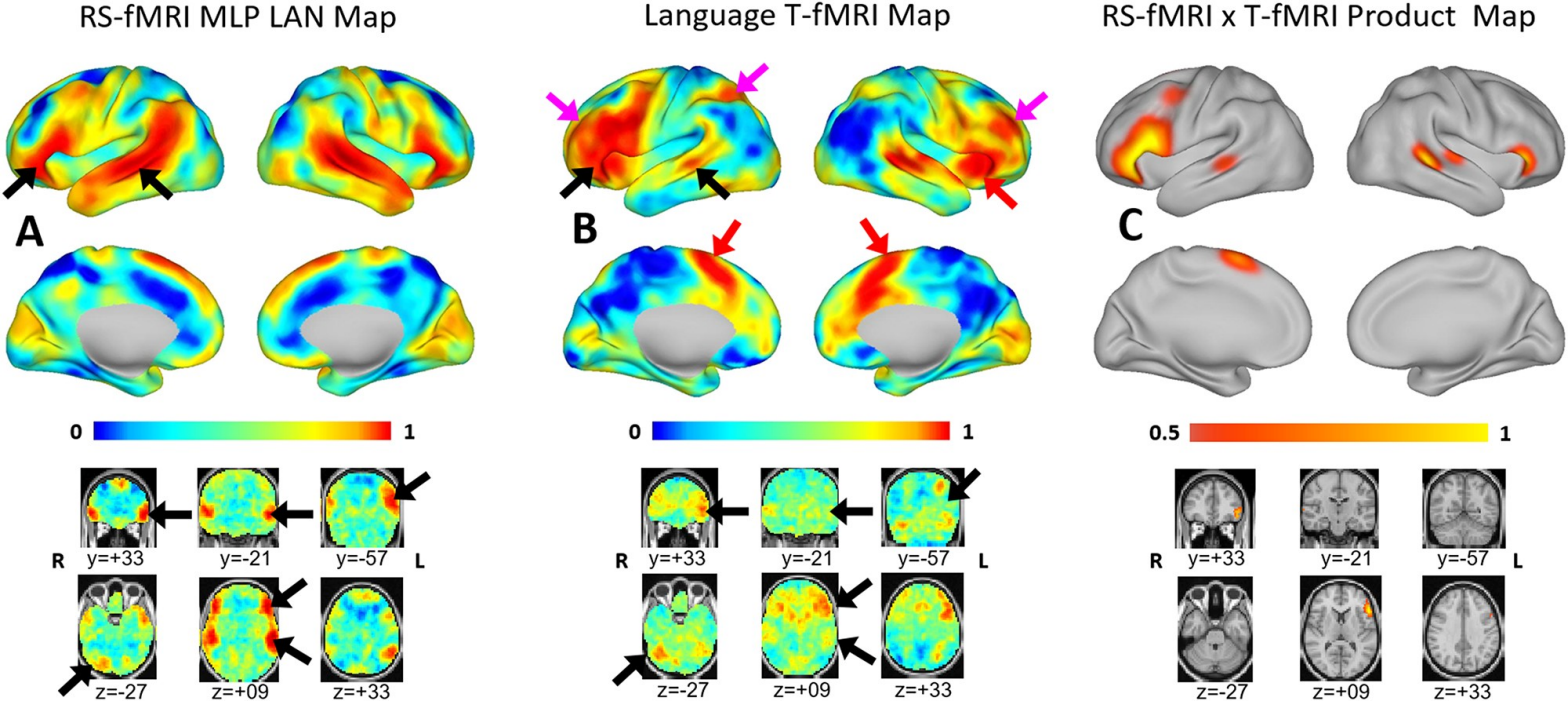
Mean language maps. Mean resting-state functional MRI and task-based functional MRI language maps displayed on the PALS B-12 inflated surface (top) and in volumetric representations with the MNI152 atlas as underlay (bottom). Note common intensity scale spanning the interval [0, 1] (see Methods (Statistical Analysis) for details concerning scale equalization). Each voxel is assigned a value in the range [0, 1] to express the likelihood of belonging to the language network. (A) Mean language map derived by MLP analysis of resting-state functional MRI data. (B) Mean task-based functional MRI response to word generation. (C) Responses common to task-based functional MRI and resting-state functional MRI computed as the product of the values shown in panels A and B, thresholded at 0.7. Black arrows point to parts of the brain specific to language function, which include, besides areas of Broca and Wernicke, the right inferior cerebellum. Red arrows point to task responses in the salience network; magenta arrows point to left intra-parietal sulcus and regions in the middle frontal gyrus bilaterally which are components of the dorsal attention network. The salience and dorsal attention networks are not specific to language.

Fig 1 shows group-level language maps derived by MLP analysis of RS-fMRI data (Fig 1A) and word-stem completion T-fMRI responses (Fig 1B). Note common intensity range spanning the interval [0, 1], imposed by response distribution matching (see Statistical Analysis, above). Both techniques generated high map values locally in inferior frontal (Broca-like) and temporal (Wernicke-like) regions (black arrows). The T-fMRI response was more extensive in the frontal regions while the RS-fMRI map was more extensive in the temporal cortex extending into the angular gyrus. A topographic concordance map, computed as the product of the T-fMRI and RS-fMRI map values, showed local maxima in Broca-like and Wernicke-like regions (Fig 1C). One point of difference is that the T-fMRI response was left-lateralized, especially in frontal areas, whereas the RS-fMRI map was more symmetric. Apart from symmetry, the major difference between methods was prominent T-fMRI (but not RS-fMRI) responses in parts of the cortex not specific to language. These areas included the rostral cingulate zone (RCZ) bilaterally and right anterior insula, which are major components of the salience system [44] (red arrows), as well as left intra-parietal sulcus and regions in the middle frontal gyrus bilaterally which are a constituent of the dorsal attention network [39] (magenta arrows). Cognitive functions of these task-general regions are considered in the Discussion.

### Consistency of RS-fMRI vs. T-fMRI maps

**Fig 2:**
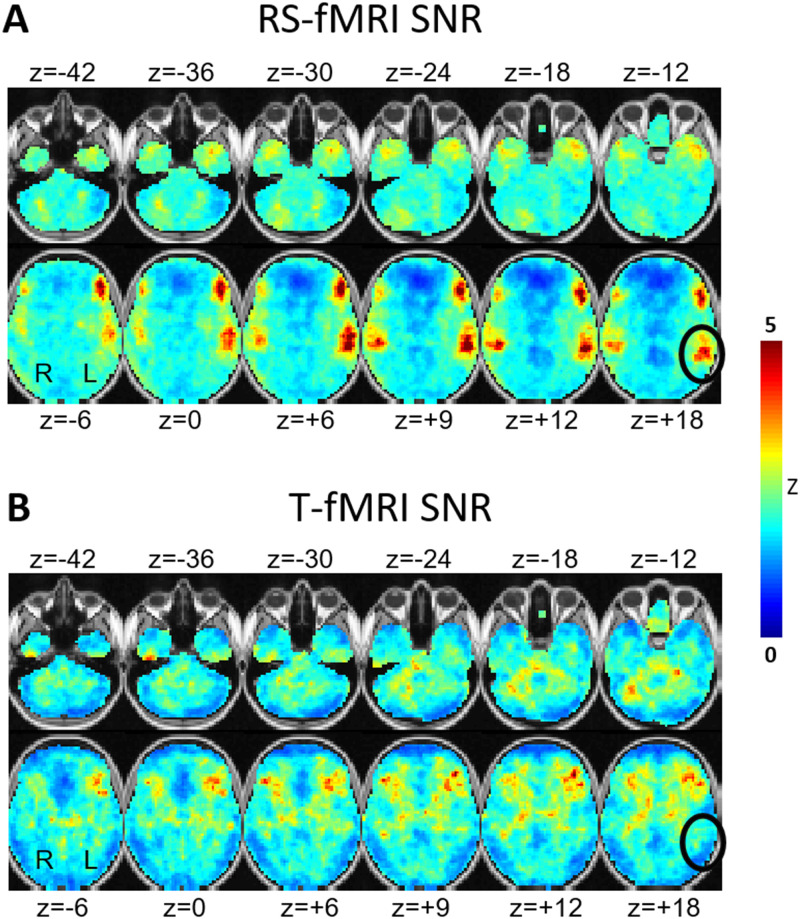
SNR maps. Task-based functional MRI and resting-state functional MRI SNR maps computed as signal-to-sampling variability ratio over individuals. High voxel values indicate consistency over subjects. (A) MLP-derived RS-MRI language RSN SNR. (B) Word-stem completion T-fMRI SNR. The black ovals indicate the same anatomic locus (Wernicke’s area) in both the MLP and T-fMRI SNR maps demonstrating markedly lower T-fMRI response consistency across individuals. The represented quantity is the voxel-wise Z-score, i.e., response mean divided by response standard deviation.

To assess response consistency over individuals, we evaluated the SNR, i.e., the voxelwise group mean response divided by standard deviation evaluated over patients. Three RS-fMRI SNR maps were obtained by dividing the available data in each patient into 3 portions, each approximately equated for T-fMRI acquisition time (S1 Fig). The average of these 3 SNR maps is shown in Fig 2; the 3 independent SNR maps are shown in Supplemental Materials. These results demonstrate greater RS-fMRI response consistency across individuals in comparison to T-fMRI. In particular, the RS-fMRI data yielded highly focal SNR maxima bilaterally in a Broca-like and Wernicke-like distribution. By comparison, T-fMRI SNR map lacked these foci and was generally more diffuse (black ovals in Fig 2).

### Match to *a priori* defined regions of interest

**Fig 3:**
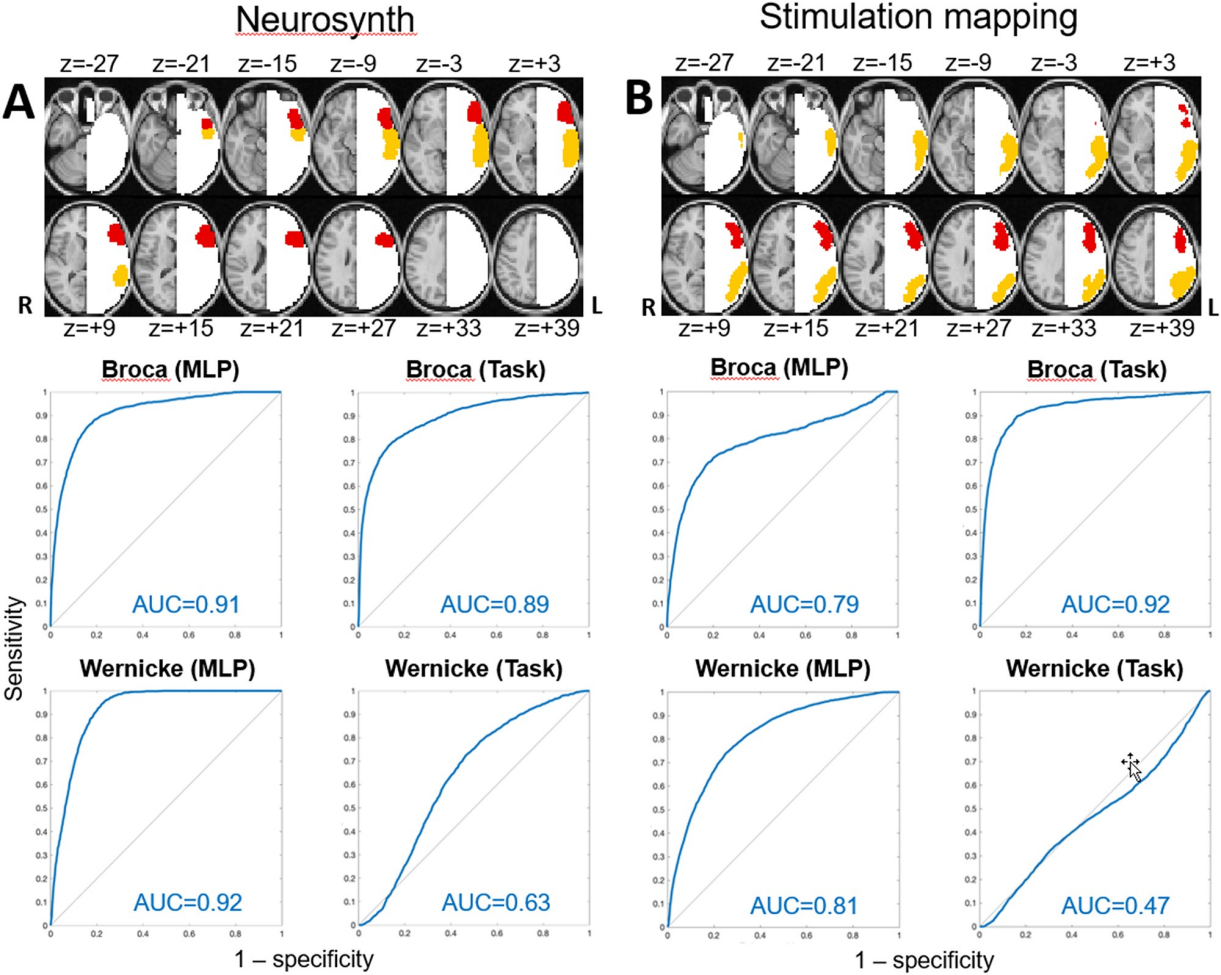
ROC curves. Broca-like (red), Wernicke-like (orange), and non-language (white) regions. Language localization receiver operating characteristic (ROC) curves (blue) for resting-state functional MRI and T-fMRI. Responses were evaluated in areas of Broca (red) and Wernicke (orange) defined either by aggregated task-based functional MRI responses (panel A) or stimulation mapping experience (panel B). Formulae defining sensitivity and specificity are given in Supplemental Materials. Area under the receiver operating characteristic curve is reported for each ROC in the lower right corner (red). Higher AUC indexes better ROC performance.

We assessed the topography of T-fMRI and RS-fMRI maps in relation to language ROIs defined on the basis of either aggregated fMRI responses to language tasks ([40]; Fig 3A) or stimulation mapping ([5]: Fig 3B). These procedures generated somewhat different representations of the areas of Broca and Wernicke (top parts of Fig 3). Nevertheless, the volumes of territory instantiating “expressive” and “receptive” functionality were approximately balanced in both cases. To construct ROC curves, "true" responses were evaluated as above-threshold response magnitude summed over the *a priori* defined ROIs (Wernicke-like or Broca-like); "false" responses were summed over regions not in either of these ROIs in the left hemisphere (see Supplemental Methods). S2 Fig reports AUC values submitted to the fast implementation of DeLong’s algorithm [43]) for evaluation of statistical significance. In most comparisons, RS-fMRI AUC exceeded T-fMRI AUC, especially in the Wernicke-like ROI. T-fMRI AUC exceeded RS-fMRI AUC only in the Broca ROI defined by stimulation mapping (AUC = 0.92 vs. 0.79 (p < 10^−6^)). In all other comparisons, RS-fMRI performance exceeded T-fMRI performance (RS-fMRI vs. T-fMRI performance: AUC = 0.91 vs. 0.89 (p < 10^−6^; Broca, Neurosynth); 0.92 vs. 0.63 (p < 10^−6^; Wernicke, Neurosynth); 0.81 vs. 0.47 (p < 10^−6^; Wernicke, stimulation mapping).

## Discussion

Prior work by our group [45] and others [30, 34, 46, 47] suggests that localizing the areas of Broca and Wernicke can be accomplished using either T-fMRI or RS-fMRI [34, 47–50]. The present results are consistent with this view (Fig 1, black arrows).

However, here we focus on the differences in maps obtained by T-fMRI vs. RS-fMRI. One difference is the greater symmetry of the localization when using RS-fMRI. Homotopic connectivity is a consistent feature of resting state networks, and is seen in both inherently symmetric networks (such as motor and vision), and also in asymmetric networks such as language. Another difference, word-stem completion T-fMRI but not RS-fMRI activated the rostral cingulate zone (RCZ) bilaterally, a region also known as the dorsal anterior cingulate (dACC), as well as the right frontal operculum (Fig 1, red arrows). These regions are components of the salience resting state network [44], alternatively known as the core executive control network [51, 52]. This functional system is recruited by a wide variety of goal-directed behaviors (e.g., see [53], fig 3; [52], fig 6; [54], fig 1). Functions attributed to the dACC include task control [55, 56], error monitoring [57] and conflict detection [58]. Also activated by T-fMRI but not RS-fMRI are regions in left superior parietal lobule and the left middle frontal gyrus bilaterally that are components of the dorsal attention network (DAN) (magenta arrows in Fig 1). The DAN responds to any task requiring directed spatial attention [39], for example, watching a screen on which task-relevant stimuli are presented. In contrast, MLP-analysis of RS-fMRI isolated regions of the brain specifically associated with language. This specificity is a design feature of the MLP, which was trained to assign to the language (LAN) RSN only parts of the brain specifically activated by language and not other, e.g., motor, attention, etc., functions [28].

The topographic differences between the maps obtained by T-fMRI vs. MLP analysis of RS-fMRI raise questions concerning what parts of the brain should be considered "eloquent," i.e., in which injury leads to functional deficits [59]. Conventionally, functions relevant to eloquence have been taken to be motor and language [60]. However, lesions of the DAN induce attentional deficits [61] and lesions of RCZ lead to a loss of motivated behaviors [62]. We suggest that a mapping procedure that distinguishes between language specific vs. attentional vs. motivational parts of the brain has value. In the current implementation of the MLP, the RCZ is assigned to the ventral attention network (VAN).

The SNR results shown in Fig 2 reveal greater inter-individual consistency of MLP analysis of RS-fMRI data in comparison to T-fMRI in the identification of language-associated cortex. This difference is especially striking in the Wernicke-like region, which is weakly activated by an expressive language task such as word-stem completion. An additional factor potentially contributing to T-fMRI response variability includes uneven performance, although all patients appeared to comply with the task. The RS-fMRI vs. T-fMRI results shown in Fig 2 are consistent with our prior study, conventionally formulated in terms of individuals, in which we found lower failure rate of RS-fMRI in comparison to T-fMRI (13% vs. 38.5%, respectively; p < 0.001) [45]. Inter-individual variability in the representation of language is unlikely to explain RS-fMRI vs. T-fMRI SNR differences, as that factor should affect both methods equally.

Fig 3 and S2 Fig compare T-fMRI vs. RS-fMRI as regards localization of the areas of Broca and Wernicke as defined *a priori*, either according to the T-fMRI literature or by stimulation mapping experience. T-fMRI AUC exceeded RS-fMRI AUC in Broca's area as defined by stimulation mapping (Fig 3B). This result is understandable as word stem completion is an expressive language task. By the same token, it understandable that RS-fMRI AUC consistently exceeded T-fMRI AUC in Wernicke's area. Regarding Neurosynth vs. stimulation mapping, in 3 of 4 cases, the T-fMRI literature-based (Neurosynth) ROIs yielded higher AUC values in comparison to the stimulation mapping-based ROIs, both for RS-fMRI and T-fMRI data (panels A vs. B in Fig 3). This result is not surprising as the MLP was trained to recover the topography of T-fMRI responses in RS-fMRI data [28]. Additionally, this outcome may reflect a relative disadvantage of stimulation mapping as speech arrest commonly is taken as the outcome measure, which arguably is a limited indicator of impaired language function [63]. Moreover, it is well established that language is represented in the frontal operculum [64], a region of the cerebral cortex not on the brain surface, and therefore, not directly accessible to stimulation mapping.

Limitations of our study include first, that it is formulated in terms of group-level analyses; hence, our results do not directly speak to the question of which technique, T-fMRI or RS-fMRI, provides the best functional localization of language in individuals. Second, our T-fMRI data were acquired using only one task whereas multiple tasks are needed to generate a more complete mapping of language function [27]. Thus, it is likely that the relatively low consistency of T-fMRI in localizing the area of Wernicke (Fig 2) could have been overcome with additional tasks emphasizing semantic operations. However, since all tasks activate task-general parts of the brain, this limitation does not compromise the point that MLP analysis of RS-fMRI localizes the representation of language more specifically than T-fMRI. Future studies with more sophisticated task designs could potentially overcome this limitation.

On the other hand, T-fMRI may the more powerful technique for determination of language lateralization: T-fMRI responses are more asymmetric, especially in Broca-like regions. However, left-lateralization of RS-fMRI LAN maps is evident in Wernicke-like regions and the right cerebellum [65]. Distorted brain anatomy in tumor patients compromises affine atlas registration; however, this issue will have affected T-fMRI and RS-fMRI equally. Minor heterogeneity of tumor histology was present (2 of 35 not glioma; Table 1); this is not expected to impact our main findings [66, 67]. Finally, we note that a definitive comparison of RS-fMRI vs. T-fMRI in terms of patient outcomes would require a prospective, multi-center, clinical trial [10]. Such a study has not yet been conducted.

## Conclusions

Our results contribute to a growing literature demonstrating that pre-surgical language mapping can be with RS-fMRI is comparable to and, in some respects, superior to T-fMRI. Indeed, our results suggest that MLP analysis of RS-fMRI data may exhibit less inter-subject variability. Whereas multiple, serially administered task paradigms are needed to activate various aspects of language functionality [27], MLP-based analysis of RS-fMRI data accomplishes the same objective simultaneously [28]. Moreover, T-fMRI activates parts of the brain that are not specific to language. Finally, we note that RS-fMRI and T-fMRI are not mutually exclusive, since it is possible to combine both types of acquisition at the analysis stage [68].

## Supporting information

S1 Fig Three resting-state functional MRI SNR maps (equated for acquisition time between resting-state vs. task-based functional MRI data) computed as signal-to-sampling variability ratio over individuals. (TIF)

S2 Fig Four panels show graphic user interface of DeLongUserInterface (https://github.com/PamixSun/DeLongUI). Blue (AUC1, receiver operating characteristic curve of resting-state fMRI derived language map), Red (AUC2, receiver operating characteristic curve of task-based fMRI derived language map).
(TIF)

S1 File (DOCX)

## References

[1] Duffau H. The necessity of preserving brain functions in glioma surgery: the crucial role of intraoperative awake mapping. World Neurosurg. 2011;76(6):525–7. 10.1016/j.wneu.2011.07.040 22251497

[2] McGirt MJ, Mukherjee D, Chaichana KL, Than KD, Weingart JD, Quinones-Hinojosa A. Association of surgically acquired motor and language deficits on overall survival after resection of glioblastoma multiforme. Neurosurgery. 2009;65(3):463–9; discussion 9–70. 10.1227/01.NEU.0000349763.42238.E9 19687690

[3] Ghinda DC, Wu JS, Duncan NW, Northoff G. How much is enough-Can resting state fMRI provide a demarcation for neurosurgical resection in glioma? Neurosci Biobehav Rev. 2018;84:245–61. 10.1016/j.neubiorev.2017.11.019 29198588

[4] Ojemann G, Ojemann J, Lettich E, Berger M. Cortical language localization in left, dominant hemisphere. An electrical stimulation mapping investigation in 117 patients. 1989. Journal of neurosurgery. 2008;108(2):411–21. 10.3171/JNS/2008/108/2/0411 18240946

[5] Sanai N, Mirzadeh Z, Berger MS. Functional outcome after language mapping for glioma resection. N Engl J Med. 2008;358(1):18–27. 10.1056/NEJMoa067819 18172171

[6] Hamberger MJ, Williams AC, Schevon CA. Extraoperative neurostimulation mapping: results from an international survey of epilepsy surgery programs. Epilepsia. 2014;55(6):933–9. 10.1111/epi.12644 24816083 PMC4057949

[7] Borchers S, Himmelbach M, Logothetis N, Karnath HO. Direct electrical stimulation of human cortex—the gold standard for mapping brain functions? Nat Rev Neurosci. 2011;13(1):63–70. 10.1038/nrn3140 22127300

[8] Silva MA, See AP, Essayed WI, Golby AJ, Tie Y. Challenges and techniques for presurgical brain mapping with functional MRI. Neuroimage Clin. 2018;17:794–803. 10.1016/j.nicl.2017.12.008 29270359 PMC5735325

[9] Haberg A, Kvistad KA, Unsgard G, Haraldseth O. Preoperative blood oxygen level-dependent functional magnetic resonance imaging in patients with primary brain tumors: clinical application and outcome. Neurosurgery. 2004;54(4):902–14; discussion 14–5. 10.1227/01.neu.0000114510.05922.f8 15046657

[10] Szaflarski JP, Gloss D, Binder JR, Gaillard WD, Golby AJ, Holland SK, et al Practice guideline summary: Use of fMRI in the presurgical evaluation of patients with epilepsy: Report of the Guideline Development, Dissemination, and Implementation Subcommittee of the American Academy of Neurology. Neurology. 2017;88(4):395–402. 10.1212/WNL.0000000000003532 28077494 PMC5272968

[11] Biswal B, Yetkin FZ, Haughton VM, Hyde JS. Functional connectivity in the motor cortex of resting human brain using echo-planar MRI. Magnetic resonance in medicine. 1995;34(4):537–41. 10.1002/mrm.1910340409 8524021

[12] Beckmann CF, DeLuca M, Devlin JT, Smith SM. Investigations into resting-state connectivity using independent component analysis. Philos Trans R Soc Lond B Biol Sci. 2005;360(1457):1001–13. 10.1098/rstb.2005.1634 16087444 PMC1854918

[13] Lee MH, Hacker CD, Snyder AZ, Corbetta M, Zhang D, Leuthardt EC, et al Clustering of resting state networks. PLoS One. 2012;7(7):e40370 10.1371/journal.pone.0040370 22792291 PMC3392237

[14] Roland JL, Griffin N, Hacker CD, Vellimana AK, Akbari SH, Shimony JS, et al Resting-state functional magnetic resonance imaging for surgical planning in pediatric patients: a preliminary experience. J Neurosurg Pediatr. 2017;20(6):583–90. 10.3171/2017.6.PEDS1711 28960172 PMC5952608

[15] Roland JL, Hacker CD, Snyder AZ, Shimony JS, Zempel JM, Limbrick DD, et al A comparison of resting state functional magnetic resonance imaging to invasive electrocortical stimulation for sensorimotor mapping in pediatric patients. Neuroimage Clin. 2019;23:101850 10.1016/j.nicl.2019.101850 31077983 PMC6514367

[16] Smith SM, Fox PT, Miller KL, Glahn DC, Fox PM, Mackay CE, et al Correspondence of the brain's functional architecture during activation and rest. Proceedings of the National Academy of Sciences of the United States of America. 2009;106(31):13040–5. 10.1073/pnas.0905267106 19620724 PMC2722273

[17] Gordon EM, Laumann TO, Gilmore AW, Newbold DJ, Greene DJ, Berg JJ, et al Precision Functional Mapping of Individual Human Brains. Neuron. 2017;95(4):791–807 e7. 10.1016/j.neuron.2017.07.011 28757305 PMC5576360

[18] Cordes D, Haughton VM, Arfanakis K, Wendt GJ, Turski PA, Moritz CH, et al Mapping functionally related regions of brain with functional connectivity MR imaging. AJNR Am J Neuroradiol. 2000;21(9):1636–44. 11039342 PMC8174861

[19] Mannfolk P, Nilsson M, Hansson H, Stahlberg F, Fransson P, Weibull A, et al Can resting-state functional MRI serve as a complement to task-based mapping of sensorimotor function? A test-retest reliability study in healthy volunteers. J Magn Reson Imaging. 2011;34(3):511–7. 10.1002/jmri.22654 21761469

[20] Dierker D, Roland JL, Kamran M, Rutlin J, Hacker CD, Marcus DS, et al Resting-state Functional Magnetic Resonance Imaging in Presurgical Functional Mapping: Sensorimotor Localization. Neuroimaging Clin N Am. 2017;27(4):621–33. 10.1016/j.nic.2017.06.011 28985933 PMC5773116

[21] Rosazza C, Aquino D, D'Incerti L, Cordella R, Andronache A, Zaca D, et al Preoperative mapping of the sensorimotor cortex: comparative assessment of task-based and resting-state FMRI. PLoS One. 2014;9(6):e98860 10.1371/journal.pone.0098860 24914775 PMC4051640

[22] Schneider FC, Pailler M, Faillenot I, Vassal F, Guyotat J, Barral FG, et al Presurgical Assessment of the Sensorimotor Cortex Using Resting-State fMRI. AJNR Am J Neuroradiol. 2016;37(1):101–7. 10.3174/ajnr.A4472 26381564 PMC7960206

[23] Schieber MH. Constraints on somatotopic organization in the primary motor cortex. Journal of neurophysiology. 2001;86(5):2125–43. 10.1152/jn.2001.86.5.2125 11698506

[24] Yeo BT, Krienen FM, Eickhoff SB, Yaakub SN, Fox PT, Buckner RL, et al Functional Specialization and Flexibility in Human Association Cortex. Cereb Cortex. 2015;25(10):3654–72. 10.1093/cercor/bhu217 25249407 PMC4598819

[25] Mennes M, Kelly C, Colcombe S, Castellanos FX, Milham MP. The extrinsic and intrinsic functional architectures of the human brain are not equivalent. Cereb Cortex. 2013;23(1):223–9. 10.1093/cercor/bhs010 22298730 PMC3513960

[26] Poldrack RA, Yarkoni T. From Brain Maps to Cognitive Ontologies: Informatics and the Search for Mental Structure. Annu Rev Psychol. 2016;67:587–612. 10.1146/annurev-psych-122414-033729 26393866 PMC4701616

[27] Benjamin CF, Walshaw PD, Hale K, Gaillard WD, Baxter LC, Berl MM, et al Presurgical language fMRI: Mapping of six critical regions. Hum Brain Mapp. 2017;38(8):4239–55. 10.1002/hbm.23661 28544168 PMC5518223

[28] Hacker CD, Laumann TO, Szrama NP, Baldassarre A, Snyder AZ, Leuthardt EC, et al Resting state network estimation in individual subjects. NeuroImage. 2013;82:616–33. 10.1016/j.neuroimage.2013.05.108 23735260 PMC3909699

[29] Poeppel D, Emmorey K, Hickok G, Pylkkanen L. Towards a new neurobiology of language. The Journal of neuroscience: the official journal of the Society for Neuroscience. 2012;32(41):14125–31. 10.1523/JNEUROSCI.3244-12.2012 23055482 PMC3495005

[30] Tanaka N, Stufflebeam SM. Presurgical Mapping of the Language Network Using Resting-state Functional Connectivity. Top Magn Reson Imaging. 2016;25(1):19–24. 10.1097/RMR.0000000000000073 26848557 PMC4833007

[31] Laumann TO, Gordon EM, Adeyemo B, Snyder AZ, Joo SJ, Chen MY, et al Functional System and Areal Organization of a Highly Sampled Individual Human Brain. Neuron. 2015;87(3):657–70. 10.1016/j.neuron.2015.06.037 26212711 PMC4642864

[32] Laumann TO, Snyder AZ, Mitra A, Gordon EM, Gratton C, Adeyemo B, et al On the Stability of BOLD fMRI Correlations. Cereb Cortex. 2017;27(10):4719–32. 10.1093/cercor/bhw265 27591147 PMC6248456

[33] Raut RV, Mitra A, Snyder AZ, Raichle ME. On time delay estimation and sampling error in resting-state fMRI. NeuroImage. 2019;194:211–27. 10.1016/j.neuroimage.2019.03.020 30902641 PMC6559238

[34] Sair HI, Yahyavi-Firouz-Abadi N, Calhoun VD, Airan RD, Agarwal S, Intrapiromkul J, et al Presurgical brain mapping of the language network in patients with brain tumors using resting-state fMRI: Comparison with task fMRI. Hum Brain Mapp. 2016;37(3):913–23. 10.1002/hbm.23075 26663615 PMC6867315

[35] Hacker CD, Roland JL, Kim AH, Shimony JS, Leuthardt EC. Resting-state network mapping in neurosurgical practice: a review. Neurosurg Focus. 2019;47(6):E15 10.3171/2019.9.FOCUS19656 31786561 PMC9841914

[36] Kamran M, Hacker CD, Allen MG, Mitchell TJ, Leuthardt EC, Snyder AZ, et al Resting-state blood oxygen level-dependent functional magnetic resonance imaging for presurgical planning. Neuroimaging Clin N Am. 2014;24(4):655–69. 10.1016/j.nic.2014.07.009 25441506 PMC4291182

[37] Baciu MV, Watson JM, Maccotta L, McDermott KB, Buckner RL, Gilliam FG, et al Evaluating functional MRI procedures for assessing hemispheric language dominance in neurosurgical patients. Neuroradiology. 2005;47(11):835–44. 10.1007/s00234-005-1431-3 16142480

[38] Power JD, Mitra A, Laumann TO, Snyder AZ, Schlaggar BL, Petersen SE. Methods to detect, characterize, and remove motion artifact in resting state fMRI. NeuroImage. 2014;84:320–41. 10.1016/j.neuroimage.2013.08.048 23994314 PMC3849338

[39] Corbetta M, Kincade JM, Ollinger JM, McAvoy MP, Shulman GL. Voluntary orienting is dissociated from target detection in human posterior parietal cortex. Nature neuroscience. 2000;3(3):292–7. 10.1038/73009 10700263

[40] Yarkoni T, Poldrack RA, Nichols TE, Van Essen DC, Wager TD. Large-scale automated synthesis of human functional neuroimaging data. Nature methods. 2011;8(8):665–70. 10.1038/nmeth.1635 21706013 PMC3146590

[41] Triantafyllou C, Hoge RD, Krueger G, Wiggins CJ, Potthast A, Wiggins GC, et al Comparison of physiological noise at 1.5 T, 3 T and 7 T and optimization of fMRI acquisition parameters. NeuroImage. 2005;26(1):243–50. 10.1016/j.neuroimage.2005.01.007 15862224

[42] DeLong ER, DeLong DM, Clarke-Pearson DL. Comparing the areas under two or more correlated receiver operating characteristic curves: a nonparametric approach. Biometrics. 1988;44(3):837–45. 3203132

[43] Sun X. X W. Fast implementation of DeLong's algorithm for comparing the areas under correlated receiver operating characteristic curves. IEEE Signal Processing Letters 2014;21(11):1389–93.

[44] Seeley WW, Menon V, Schatzberg AF, Keller J, Glover GH, Kenna H, et al Dissociable intrinsic connectivity networks for salience processing and executive control. The Journal of neuroscience: the official journal of the Society for Neuroscience. 2007;27(9):2349–56. 17329432 10.1523/JNEUROSCI.5587-06.2007 PMC2680293

[45] Leuthardt EC, Guzman G, Bandt SK, Hacker C, Vellimana AK, Limbrick D, et al Integration of resting state functional MRI into clinical practice—A large single institution experience. PLoS One. 2018;13(6):e0198349 10.1371/journal.pone.0198349 29933375 PMC6014724

[46] Parker Jones O, Voets NL, Adcock JE, Stacey R, Jbabdi S. Resting connectivity predicts task activation in pre-surgical populations. Neuroimage Clin. 2017;13:378–85. 10.1016/j.nicl.2016.12.028 28123949 PMC5222953

[47] Tie Y, Rigolo L, Norton IH, Huang RY, Wu W, Orringer D, et al Defining language networks from resting-state fMRI for surgical planning—a feasibility study. Hum Brain Mapp. 2014;35(3):1018–30. 10.1002/hbm.22231 23288627 PMC3683367

[48] Lemee JM, Berro DH, Bernard F, Chinier E, Leiber LM, Menei P, et al Resting-state functional magnetic resonance imaging versus task-based activity for language mapping and correlation with perioperative cortical mapping. Brain Behav. 2019;9(10):e01362 10.1002/brb3.1362 31568681 PMC6790308

[49] Branco P, Seixas D, Deprez S, Kovacs S, Peeters R, Castro SL, et al Resting-State Functional Magnetic Resonance Imaging for Language Preoperative Planning. Front Hum Neurosci. 2016;10:11 10.3389/fnhum.2016.00011 26869899 PMC4740781

[50] Lu J, Zhang H, Hameed NUF, Zhang J, Yuan S, Qiu T, et al An automated method for identifying an independent component analysis-based language-related resting-state network in brain tumor subjects for surgical planning. Sci Rep. 2017;7(1):13769 10.1038/s41598-017-14248-5 29062010 PMC5653800

[51] Dosenbach NU, Fair DA, Miezin FM, Cohen AL, Wenger KK, Dosenbach RA, et al Distinct brain networks for adaptive and stable task control in humans. Proceedings of the National Academy of Sciences of the United States of America. 2007;104(26):11073–8. 10.1073/pnas.0704320104 17576922 PMC1904171

[52] Dosenbach NU, Visscher KM, Palmer ED, Miezin FM, Wenger KK, Kang HC, et al A core system for the implementation of task sets. Neuron. 2006;50(5):799–812. 10.1016/j.neuron.2006.04.031 16731517 PMC3621133

[53] Hugdahl K, Raichle ME, Mitra A, Specht K. On the existence of a generalized non-specific task-dependent network. Front Hum Neurosci. 2015;9:430 10.3389/fnhum.2015.00430 26300757 PMC4526816

[54] Nelson SM, Dosenbach NU, Cohen AL, Wheeler ME, Schlaggar BL, Petersen SE. Role of the anterior insula in task-level control and focal attention. Brain Struct Funct. 2010;214(5–6):669–80. 10.1007/s00429-010-0260-2 20512372 PMC2886908

[55] Kolling N, Wittmann MK, Behrens TE, Boorman ED, Mars RB, Rushworth MF. Value, search, persistence and model updating in anterior cingulate cortex. Nature neuroscience. 2016;19(10):1280–5. 10.1038/nn.4382 27669988 PMC7116891

[56] Shackman AJ, Salomons TV, Slagter HA, Fox AS, Winter JJ, Davidson RJ. The integration of negative affect, pain and cognitive control in the cingulate cortex. Nat Rev Neurosci. 2011;12(3):154–67. 10.1038/nrn2994 21331082 PMC3044650

[57] Neta M, Miezin FM, Nelson SM, Dubis JW, Dosenbach NU, Schlaggar BL, et al Spatial and temporal characteristics of error-related activity in the human brain. The Journal of neuroscience: the official journal of the Society for Neuroscience. 2015;35(1):253–66. 25568119 10.1523/JNEUROSCI.1313-14.2015 PMC4287146

[58] Carter CS, van Veen V. Anterior cingulate cortex and conflict detection: an update of theory and data. Cogn Affect Behav Neurosci. 2007;7(4):367–79. 10.3758/cabn.7.4.367 18189010

[59] Kahn E, Lane M, Sagher O. Eloquent: history of a word's adoption into the neurosurgical lexicon. Journal of neurosurgery. 2017;127(6):1461–6. 10.3171/2017.3.JNS17659 29027861

[60] Spetzler RF, Martin NA. A proposed grading system for arteriovenous malformations. Journal of neurosurgery. 1986;65(4):476–83. 10.3171/jns.1986.65.4.0476 3760956

[61] Baldassarre A, Ramsey L, Hacker CL, Callejas A, Astafiev SV, Metcalf NV, et al Large-scale changes in network interactions as a physiological signature of spatial neglect. Brain. 2014;137(Pt 12):3267–83. 10.1093/brain/awu297 25367028 PMC4240302

[62] Cohen RA, Kaplan RF, Zuffante P, Moser DJ, Jenkins MA, Salloway S, et al Alteration of intention and self-initiated action associated with bilateral anterior cingulotomy. J Neuropsychiatry Clin Neurosci. 1999;11(4):444–53. 10.1176/jnp.11.4.444 10570756

[63] Brennan NP, Peck KK, Holodny A. Language Mapping Using fMRI and Direct Cortical Stimulation for Brain Tumor Surgery: The Good, the Bad, and the Questionable. Top Magn Reson Imaging. 2016;25(1):1–10. 10.1097/RMR.0000000000000074 26848555 PMC4966674

[64] Friederici AD, Gierhan SM. The language network. Curr Opin Neurobiol. 2013;23(2):250–4. 10.1016/j.conb.2012.10.002 23146876

[65] Joliot M, Tzourio-Mazoyer N, Mazoyer B. Intra-hemispheric intrinsic connectivity asymmetry and its relationships with handedness and language Lateralization. Neuropsychologia. 2016;93(Pt B):437–47. 10.1016/j.neuropsychologia.2016.03.013 26988116

[66] Briganti C, Sestieri C, Mattei PA, Esposito R, Galzio RJ, Tartaro A, et al Reorganization of functional connectivity of the language network in patients with brain gliomas. AJNR Am J Neuroradiol. 2012;33(10):1983–90. 10.3174/ajnr.A3064 22555573 PMC7964610

[67] Hart MG, Romero-Garcia R, Price SJ, Suckling J. Global Effects of Focal Brain Tumors on Functional Complexity and Network Robustness: A Prospective Cohort Study. Neurosurgery. 2018. 10.1093/neuros/nyy378 PMC6520100 30137556

[68] Wang D, Buckner RL, Fox MD, Holt DJ, Holmes AJ, Stoecklein S, et al Parcellating cortical functional networks in individuals. Nature neuroscience. 2015;18(12):1853–60. 10.1038/nn.4164 26551545 PMC4661084

