# Supplementary figures and images for: Mapping language function with task-based vs. resting-state functional MRI
Source: PLoS One. 2020 Jul 31;15(7):e0236423. doi: 10.1371/journal.pone.0236423 (PMC7394427; doi:10.1371/journal.pone.0236423)

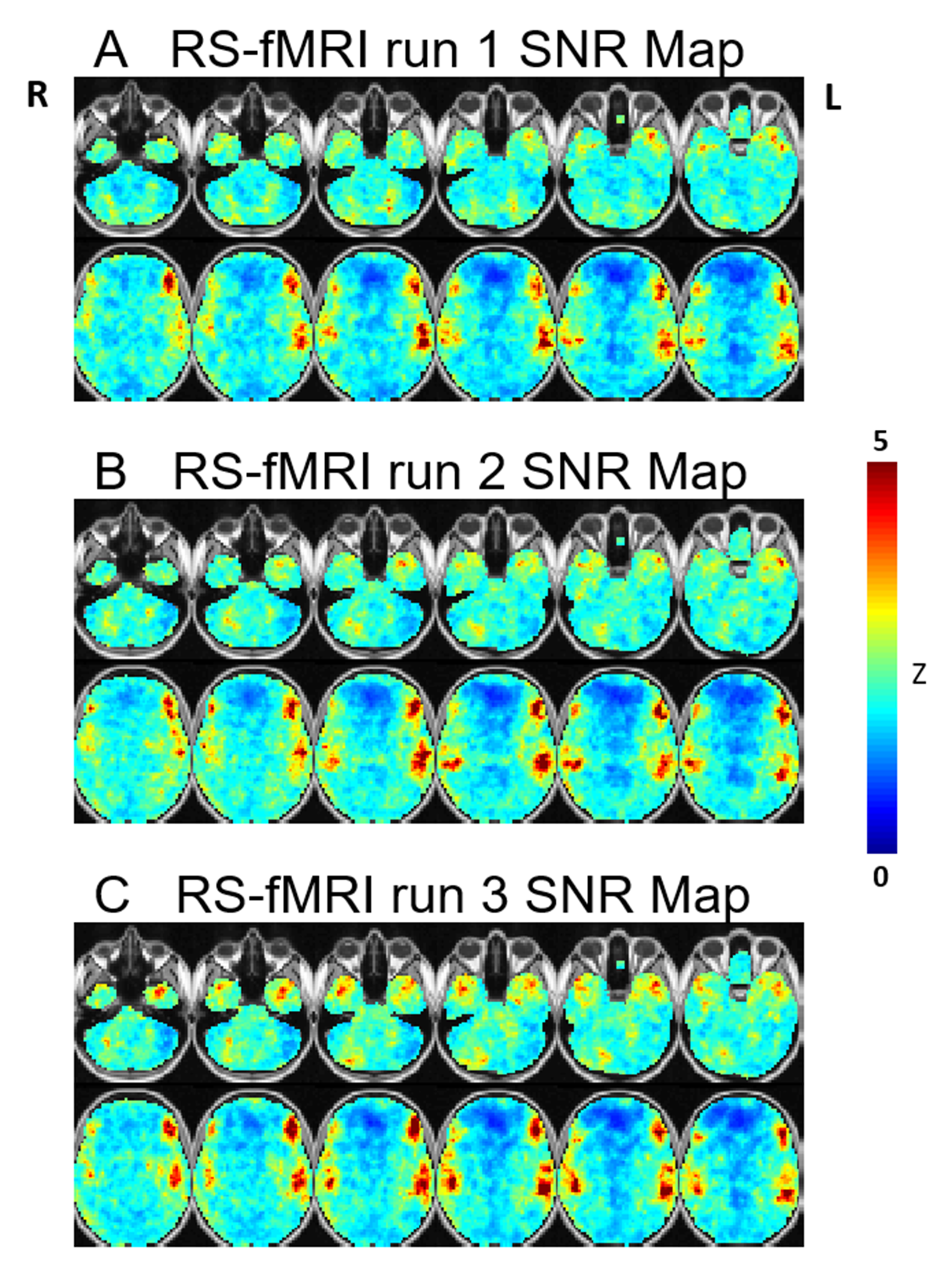

Supplement: S1 Fig — (TIF) [file pone.0236423.s001.tif]

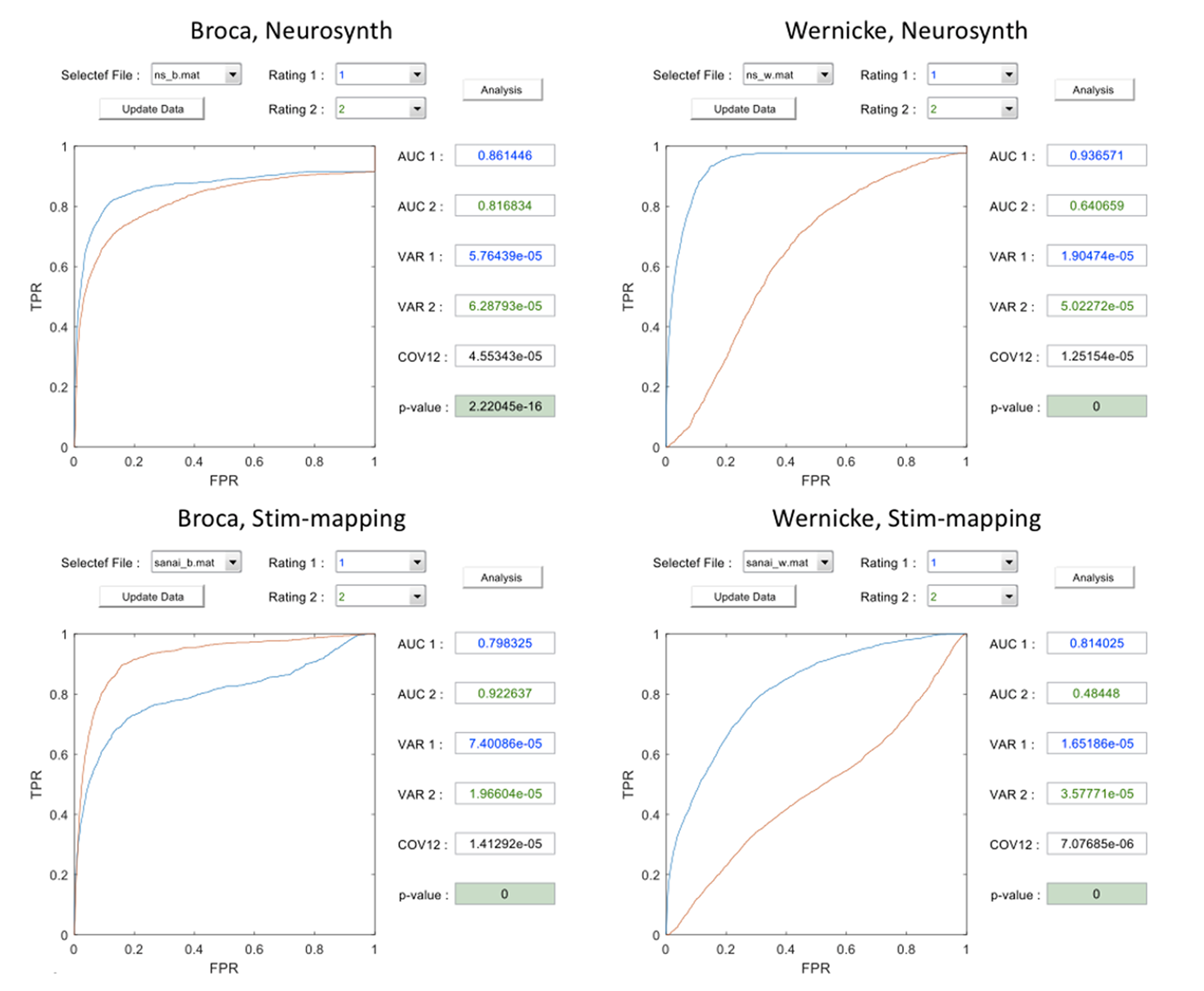

Supplement: S2 Fig — Blue (AUC1, receiver operating characteristic curve of resting-state fMRI derived language map), Red (AUC2, receiver operating characteristic curve of task-based fMRI derived language map). (TIF) [file pone.0236423.s002.tif]
