# Supplementary material for: Mapping language function with task-based vs. resting-state functional MRI
Source: PLoS One. 2020 Jul 31;15(7):e0236423. doi: 10.1371/journal.pone.0236423 (PMC7394427; doi:10.1371/journal.pone.0236423)
Supplement: S1 File — (DOCX) [file pone.0236423.s003.docx]

# **Supporting Information**

**Preprocessing**

Preprocessing steps included compensation for slice dependent time shifts, elimination of systemic odd-even slice intensity differences due to interleaved acquisition, and rigid body correction for head movement within and across runs. Atlas transformation was achieved by composition of affine transforms connecting the fMRI volumes with the T2-weighted and then to the T1-weighted MP-RAGE structural images, and finally to the atlas space images, resulting in a volumetric time series in (3mm)^3^ voxel atlas space.

Additional preprocessing in preparation for RS-fMRI analysis included spatial smoothing (6 mm full width half maximum Gaussian blur in each direction), voxelwise removal of linear trends over each run, and temporal low pass filtering retaining frequencies <0.1 Hz. Spurious variance was reduced by regression of nuisance waveforms derived from head motion correction and extraction of the time series from regions of white matter and CSF. The whole brain (“global”) signal was included as a nuisance regressor (66, 67). Frame censoring was performed to minimize the impact of head motion on the correlation results (35). Frames in which the whole brain root mean square change in voxel intensity exceeded 0.5% were excluded from the functional connectivity computations (68).

**Standard View of Language Representation on the Cerebral Cortex**

Language is widely understood to be represented primarily in two areas of the cerebral cortex: Broca’s area is located in inferior frontal cortex (roughly, Brodmann areas 44 and 45) and frontal operculum (61); Broca's area is required for fluid performance of accurate phonemic or semantic tasks (69); Wernicke's area extends over potions of temporal and parietal cortex and is essential for understanding written or spoken language (70). These functions are left lateralized in the great majority of humans (71). Allowing that the preceding account is oversimplified (27, 72, 73), the Broca-Wernicke model provides a useful basis on which to define regions of interest (ROIs) for purposes of evaluating T-fMRI vs. RS-fMRI in the context of pre-surgical language mapping (74).

**Definition of Broca and Wernicke ROIs on the Basis of Aggregated T-fMRI**.

Neurosynth (37) identified 107 studies (as of November, 2018) contributing coordinates in Talairach atlas space, each coordinate associated with a Z-score corresponding to the null hypothesis of equally likely activation anywhere in the brain. The returned association map (thresholded at Z > 3.7 by Neurosynth) was passed through smoothing and clustering operations (see below), ultimately yielding Broca- and Wernicke-like ROIs in volumetric atlas space (**Figure 3A**).

The following steps were taken to obtain Broca- and Wernicke-like regions of interest (ROIs) starting with a “language comprehension” association map (units = Z-score) generated by Neurosynth in (2mm)^3^ MNI152 atlas space:

1. Gaussian smooth using a kernel of 1mm full width at half maximum (FWHM) in each cardinal direction.
2. Threshold at Z-score > 3.7.
3. Retain two largest clusters → Initial Broca- and Wernicke-like regions in the left hemisphere.
4. Gaussian smooth using a 3mm FWHM kernel in each cardinal direction.
5. Resolve overlapping clusters into two disjoint ROIs by assigning multiply labeled voxels to the ROI with the nearest center of mass.

**Definition of Broca and Wernicke ROIs on the Basis of Stimulation Mapping (2)**.

All loci with were identified on the basis of anatomical landmarks (adjacent gyri and sulci) and transferred to the inflated standard PALS-B12 atlas surface. Broca- and Wernicke-like loci were encircled by closed borders and the resulting surface parcels were projected 1.5mm above and below the mean PALS-B12 mid-thickness surface. These regions then were dilated by two (3mm)^3^ voxels to generate ROIs in volumetric atlas space (**Figure 3B**).

**Image Computation and Visualization Software**

| **Software/Data** | **Version/Year** | **Publisher** | **City/State** | **Open Source** |
| --- | --- | --- | --- | --- |
| AFNI_17.3.03 | 17.3.03, 64-bit, 2017 | NIMH/NIH | Bethesda, MD | Yes |
| Caret | v5.65 for Linux, 2012 | Van Essen Lab, Washington University | St. Louis, MO | Yes |
| Connectome Workbench | v1.2.3, 2016 | Van Essen Lab, Washington University | St. Louis, MO | Yes |
| PALS-B12 atlas | 2005 | Van Essen Lab, Washington University | St. Louis, MO | Yes |

**Formulae used to compute signal-to-sampling variability ratio (SNR):**

The following equation describes how the SNR was computed:

$$SSR_{v}={\mu_{v}}/{\sigma_{v}}$$

where $\mu_{v}$ is mean value of the language response map voxel $v$ and $\sigma_{v}$ is the standard deviation.

**Formulae used to compute receiver operating characteristic (ROC) curves:**

The following prescribes the algebra used to construct receiver operator characteristic (ROC) curves shown in Figure 3.

Define $MAP_{LAN}$: Language ROIs (voxels) generated by two reference methods, literature atlas coordinates or stimulation mapping

Define $MAP_{fMRI}$: Language maps generated either by T-fMRI or RS-fMRI

Define $Map_{fMRI}^{C}$: Areas (in the left hemisphere) not included in $MAP_{fMRI}$

Define $Map_{LAN}^{C}$: Areas (in the left hemisphere) not included in $MAP_{LAN}$

True positive: $TP=Map_{fMRI}\cap Map_{LAN}$

False positive: $FP=Map_{fMRI}\cap Map_{LAN}^{C}$

True negative: $TN=Map_{fMRI}^{C}\cap Map_{LAN}^{C}$

False negative: $FN=Map_{fMRI}^{C}\cap Map_{LAN}$

Sensitivity: ${TP}/{(TP+FN})$

Specificity: ${TN}/{(TN+FP)}$
